# Mud Crab Genus *Scylla* Broodstock Culture: Current Challenges and the Potential of a Hybrid Specific Pathogen Free Approach

**DOI:** 10.21315/tlsr2026.37.3.1

**Published:** 2026-10-15

**Authors:** Sor Chin Siang, Chiar Yu Shan, Hanafiah Fazhan, Khor Waiho

**Affiliations:** 1 Higher Institution Centre of Excellence (HICoE), Institute of Tropical Aquaculture and Fisheries, Universiti Malaysia Terengganu, 21030 Kuala Nerus, Terengganu, Malaysia; 2 Sayap Jaya Sdn Bhd., No. 1-2Ab, Jalan Desa 1/3. Desa Aman Puri, 52100 Kuala Lumpur, Malaysia

**Keywords:** *Scylla paramamosain*, Specific Pathogen Free, Prophylactic, Broodstock Management, Hatchery, Scylla paramamosain, Specific Pathogen Free, Profilaktik, Pengurusan Induk, Hatcheri

## Abstract

Mud crab (genus *Scylla*) farming forms an integral part of coastal aquaculture in Asia region, with their cultivation supporting coastal livelihoods, driving economic growth through their high market demand and promoting mangrove forest conservation and environmental sustainability via mangrove ecosystem integration. However, instability in the production chain, especially the early stages from broodstock cultivation to larval rearing and crablet, promoted the continued reliance on wild-caught broodstock. Such reliance on wild-caught broodstock introduces significant biosecurity risks, particularly through pathogen transmission and inconsistent reproductive performance. Current management selects mature females based on size and ovarian development, followed by acclimation and prophylactic formalin baths to mitigate bacterial and fungal infections. This review examines the biosecurity risks of relying on wild broodstock and proposes a practical “hybrid” approach that combines the innate immune resilience of wild populations with Specific Pathogen Free (SPF)-like conditions to enhance disease resistance, reproductive success and overall production sustainability. Nutritional strategies employ high-protein feeds, such as fish and bivalves, to enhance ovarian maturation, but untreated wild-caught feeds heighten pathogen transmission risks. By quarantine, disinfection and microbiome management protocol, pathogen load can be reduced while innate immunity can be improved.

## Abstrak

Kaedah penternakan secara ekstensif amat penting dalam akuakultur tropika, kerana ia menyokong mata pencarian penduduk pesisir, memacu pertumbuhan ekonomi melalui permintaan pasaran, dan menggalakkan pemuliharaan hutan bakau serta kelestarian alam sekitar melalui integrasi ekosistem bakau. Namun, ketidakstabilan dalam rantaian pengeluaran, terutamanya pada peringkat awal seperti pemeliharaan induk, penetasan larva dan pembesaran anak ketam telah mengurangkan kecekapan dalam penghasilan ketam dewasa bersaiz pasaran. Kebergantungan terhadap induk tangkapan liar membawa risiko biosekuriti yang ketara, terutamanya melalui transmisi patogen dan prestasi reproduktif yang tidak konsisten. Pengurusan semasa memilih induk betina matang berdasarkan saiz dan kematangan ovari, diikuti dengan proses aklimatisasi dan rendaman formalin secara profilaktik untuk mengurangkan jangkitan bakteria dan kulat. Strategi pemakanan menggunakan makanan berprotein tinggi seperti ikan dan moluska bercangkerang bagi merangsang pematangan ovari, namun penggunaan makanan tangkapan liar yang tidak dirawat meningkatkan risiko transmisi patogen. Melalui protokol kuarantin, pembasmian kuman, pengurusan mikrobiom dan beban patogen dapat dikurangkan, manakala imuniti semula jadi dapat dipertingkatkan.

**HIGHLIGHTS**

- Mud crab (genus Scylla) farming forms an integral part of coastal aquaculture in Asia region. Instability in the production chain promoted the continued reliance on wild-caught broodstock.
- Nutritional strategies employ high-protein feeds to enhance ovarian maturation, but untreated wild-caught feeds heighten pathogen transmission risks.

## INTRODUCTION

Mud crabs of the genus *Scylla* are predominantly found in association with mangrove ecosystems, where their habitats encompass intertidal and subtidal muddy and sandy-muds area (Le Vay *et al*. 2007; Holme *et al*. 2009). Mud crab possess significant market value and commercially important for fishermen and coastal communities throughout their habitats (Petersen *et al*. 2013; Bhuiyan *et al*. 2021). Over the past few decades, mud crab has become established as a pivotal species in the aquaculture sector, particularly in Southeast Asia where interest in the aquaculture of *Scylla* species has been documented in tropical Asia since the 1970s (Bir 2016; Syafaat *et al*. 2021). However, instability in the production chain, from broodstock cultivation to larval rearing and the growth of market-sized adult crabs, remains a significant challenge in mud crab farming due to disease outbreaks and the biosecurity risks associated with relying on wild-caught broodstock (Pattiasina *et al*. 2016; Waiho *et al*. 2018 Liew *et al*. 2023). The objective of this review is to examine the biosecurity risks associated with relying on wild-caught broodstock and to outline a practical ‘hybrid’ approach that combines natural immune resilience with SPF-like conditions to improve disease resistance and production sustainability.

## CORE MANAGEMENT PRACTICES IN MUD CRAB AQUACULTURE

### Broodstock Selection

Most of the mud crab broodstock is sourced from wild-caught individuals (Table 1), which poses a high risk of pathogen transmission and requires rigorous disease control measures (Shelley 2008; Lovatelli *et al*. 2025). Broodstock management is crucial for enhancing reproduction and genetic improvements, allowing the entire production chain to achieve sustained efficiency and optimal productivity (Duncan *et al*. 2013). The implementation of broodstock management protocols and nutritional strategies significantly influences reproductive parameters, including fecundity, hatching rate, larvae survival rate and growth to maturity (Bromage 1998). The cultivation of optimal broodstock yields high-quality eggs, generally characterised by high fertilisation rates, eye stage development and hatchability.

Mud crab are collected from their natural habitat using methods such as, baited traps, gill nets and handpicking, often sourced directly from fishermen (Butcher *et al*. 2012; Funde *et al*. 2013; Broadhurst *et al*. 2014; Azra & Ikhwanuddin 2016; Fitri *et al*. 2017). After acquiring mud crabs from local fishermen, mature females weighing more than 200 g with all limbs intact and exhibiting fourth-stage ovarian maturation, identified by deep orange-red ovaries that fully occupy the body cavity and are clearly visible when a torchlight is shone through the carapace in a darkened room (Azra & Ikhwanuddin 2016; Hajisamae *et al*. 2025; Lovatelli *et al*. 2025). The broodstocks then undergo a two-day acclimation period before being placed in individual holding system (Lovatelli *et al*. 2025).

### Prophylactics Treatment

Formalin is commonly used as a prophylactic agent to eliminate the indigenous bacterial flora of mud crab broodstock. Prophylactic treatment of 150 ppm formalin solution for 30 min is usually practiced (Hamasaki & Hatai 1993; Azra & Ikhwanuddin 2016; Leal *et al*. 2018a; Lovatelli *et al*. 2025). Post-prophylactic treatment, the broodstock are transferred to individual holding tanks with a substrate of fine sand at the bottom, equipped with flowing water and aeration for berried cultivation (Lovatelli *et al*. 2025).

During spawning stage, recent research indicate that mud crab broodstock exhibits a strong preference for fine sand (< 70 micron) over coarse sand or bare tank bottom, therefore the presence of treated sand is critical not only facilitates the spawning, but also significantly reduce potential external pathogen infection (Fazhan *et al*. 2022). Once berried broodstock hatched, prophylactic formalin treatments applied to active zoea 1 for approximately 10 sec to mitigate fungal infection and reduce bacterial load (Hamasaki & Hatai 1993). This is a standard protocol in the management of mud crab broodstock for controlling infectious diseases that impact reproduction performance.

### Nutritional Management

The nutritional and feeding strategies for mud crab broodstock rely on wide variety of feeds, including trash fish, squid, mussel meat and blood cockle (Millamena & Quinitio 2000; Keenan & Blackshaw 2011; Azra & Ikhwanuddin 2016). The primary focus in designing dietary strategies for broodstock is gonadal maturation, with nutrient profiles such as high protein and amino acids like alanine, cystine and leucine (Keenan & Blackshaw 2011; Early Septiningsih & Herlinah 2015). These feeds, commonly sourced from untreated wild catches, pose a potential risk for disease and pathogen transmission. Frequently identified agents of transmission pathogens within genus *Vibrio* included *V. parahaemolyticus, V. vulnificus, V. cholerae*, *V. alginolyticus*, *V. splendidus* and *V. haryeiyi* (Austin & Austin 2007; Jones & Oliver 2009; Jithendran *et al*. 2010; Deng *et al*. 2019; Cheng *et al*. 2020).

## DISEASES MANAGEMENT

### Mud Crab Diseases

It poses a critical risk of disrupting the immune defences of the host through direct host-pathogen interactions or worsening the situation via horizontal gene transfer among the vibrios to the host (Jithendran *et al*. 2010; Le Roux & Blokesch 2018; Deng *et al*. 2019 Zhang *et al*. 2020). It also increases susceptibility to fungal infections, including those caused by *Lagenidium* spp., which induce egg mortality and larval mycosis in shrimp and crab (Jithendran *et al*. 2010).

Mud crab rely on their innate immune system to counteract pathogen, such risk may result in the immune system prioritising pathogen resistance over reproductive performance (Chen & Wang 2019). The application of formalin as a disinfectant prior to broodstock captivity culture may affect immune response ability due to its electrophilic character, which enables interaction with several biological molecules, such as proteins, DNA and RNA (McDonnell & Russell 1999; Leal *et al*. 2018b). Formalin not only eliminates native bacteria cells from the carapace of wild-caught broodstock but may also harm the broodstock’s innate immune system, such as disrupting antimicrobial peptide that enhance phagocytosis and Antimicrobial Peptide (AMP) synthesis (Denyer & Stewart 1998). The potential risk on over exposure to formalin can lead to oxidative stress, resulting in imbalance in Reactive Oxygen Species (ROS) production and antioxidant defence (Cheng *et al*. 2021).

Bacterial infection in mud crabs can suppress hemocyte activity, allowing pathogens to invade (Zhou *et al*. 2018). Hemocytes are the key immune cell in crustaceans and play a crucial role in pathogen defence through phagocytosis, melanisation, phenoloxidase activity and regulation of immune gene expression (Mangkalanan *et al*. 2014; Kankamol & Salaenoi 2018). The stimulation of exosome secretion and modulation of anti-lipopolysaccharide factor (ALF) expression can impair immune recognition and binding to pathogens, potentially weakening the host’s ability to initiate an effective defense against infections (Sun *et al*. 2022).

### Specific Pathogen Free (SPF) Approach

The cultivation of broodstock mud crab in a closed recirculating system that meets the Specific Pathogen Free (SPF) requirements potentially providing a novel approach to optimise its performance. The remarkable success of SPF broodstock and larval of *Penaeus monodon* and *Litopenaeus vannamei* in the industry adds tremendous economic value (Lightner *et al*. 2009; Wyban 2009).

Unlike vertebratas, crustaceans lack an adaptive immune system and do not produce antibodies (Cerenius *et al*. 2010; Wang *et al*. 2014), yet they can still transmit pathogen to their offspring, leading to high morbidity and mortality rates. The development of SPF broodstock and larvae for crustacean like *P. monodon* and *L. vannamei* fundamentally follows strict biosecurity protocols for two consecutive years at a certified rearing facility to ensure the absence of specific pathogen listed by the Office International desEpizooties and is maintained on biosecure feeds (Lightner 2007; Lightner *et al*. 2009; Murray *et al*. 2022). Complying with high-level biosecurity protocols, as documented for SPF, not only safeguards pathogen exclusion but also contributes to genetic improvement (Lightner *et al*. 2009). Genetic characterisation involves Specific Pathogen Resistant (SPR) and Specific Pathogen Tolerant (SPT), where SPR refers to individuals genetically selected to resist specific pathogens, while SPT indicates that they may still become infected but exhibit minimal symptoms or performance loss (Alday-Sanz 2018).

### Biosecure Transitional Broodstock and Hatchery System

Nevertheless, practical gaps exit in achieving SPF status for mud crab broodstock and larvae. Unlike shrimp, where SPF programs are well-established, the research and development of SPF mud crab remain limited (Alday-Sanz *et al*. 2018). Enforcing biosecurity is challenging due to their agonistic and cannibalistic behaviour, which makes them more difficult to manage in confined system compared to shrimp (Mirera & Moksnes 2013; Junaidi Zakaria & Agustina Saragih 2021; Liew *et al*. 2023).

Additionally, the dietary needs of mud crab also increase the risk of pathogen introduction, as artificial sterile feeds for *Scylla* spp. are not readily available. The primary consideration in establishing and maintaining SPF is the significant financial investment required due to several key factors, including infrastructure development, comprehensive biosecurity measures, high-quality water treatment systems, continuous health monitoring and disease screening. The development of SPF shrimp broodstock involves domestication, selective breeding, rigorous quarantine procedures and a highly sterilised environment (Lightner 2007; Lightner *et al*. 2009). The most critical aspect of this process is the strict prevention of pathogen exposure to ensure the health and biosecurity of the broodstock. By applying these criteria, the broodstock potentially miss out on immune priming and microbiome diversity, possibly leaving the offspring or the broodstock less equipped to combat real-world disease during the grow-out phase (Pope *et al*. 2011). Juveniles of mud crab need more space compared to shrimp due to their natural living behaviours, including territoriality and cannibalism, as they live a benthic lifestyle (Mirera & Moksnes 2013; Hannan *et al*. 2024). Moreover, their grow-out process is typically carried out in a diverse range of uncontrolled farms or earthen pond where environmental conditions and biosecurity measures may be challenging to regulate (Ut *et al*. 2007; Mirera & Moksnes 2015). Wild-caught crabs, naturally resilient from their environment, probably have a more robust innate immune response, even if they possess pathogens (Zhang *et al*. 2020).

To bridge the gaps, a hybrid approach involving the design of SPF-like conditions for wild-caught mud crab broodstock could balance the natural immune resilience of wild population with the disease-free advantages of SPF systems and biosecure feed. The breeding process would start by leveraging the benefits of wild-caught mud crabs, which would likely possess robust innate immunity derived from diverse microbiomes acquired through natural pathogen exposure, and transitioning them to a controlled, pathogen-free environment that mimics SPF conditions (Jiang *et al*. 2023).

This resilience might also contribute to a reduction in the time required for breeding SPF broodstock, as wild-caught crabs would have a robust immune system that could enhances their adaptability and resistance to pathogens in SPF-like conditions. As a result, the offspring might exhibit improved growth rates and higher disease resistance (Hamasaki & Hatai 1993). Based on broodstock aquaculture principles, capture and initial quarantine could be used in this hybrid approach, which would isolate crabs to assess their health, including PCR screening to identify microbial presence and potential virulence factors. The wild-caught broodstock would be placed in a quarantine tank with SPF-like conditions, such as filtered and UV-treated seawater, with water salinity of 32 psu–35 psu, pH of 7.5–8.5, and alkalinity of 120 mg/L–180 mg/L, within a high-biosecurity-level hatchery (Lovatelli *et al*. 2025).

Since wild-caught broodstock often host pathogen, to minimise disease threats, after the initial quarantine, they can be disinfected with formalin as practice in documented mud crab aquaculture protocols, which might also help achieve SPF-like conditions (Hamasaki & Hatai 1993; Jithendran *et al*. 2010; Keenan & Blackshaw 2011). The formalin exposure duration and concentration would need to be carefully assessed to minimise potential stress, which could impair the immune system while still effectively clearing latent infections. A detachable microbiome test would be conducted with rigorous screening and thorough risk assessment after the disinfectant treatment. Comprehensive screening for White Spot Syndrome Virus (WSSV), Mud Crab Reovirus (MCRV) and *Vibrio* species would be performed using PCR (Jithendran *et al*. 2010). A follow-up screening would assess pathogen clearance and confirm pathogen-free status prior to the initiation of breeding. A post-treatment recovery period could be implemented, allowing the crab to recover in a well-oxygenated, biosecurity water with nutritionally enriched feeds.

The treated broodstock individuals would be transitioned to biosecure holdings that mimic SPF conditions, utilising screened natural feeds and potential sterilisation methods such as ozone treatment, UV radiation or high-temperature processing. Strict biosecurity measures, including footbaths, equipment disinfection and restricted access, would be implemented to prevent recontamination. The broodstock gut microbiome would be stimulated with probiotics, for example, *Lactobacillus* or *Bacillus* spp., to enhance haemocyte activity and improve infection response (Wu *et al*. 2014). SPF-like water conditions and biosecurity feeding protocol would be maintained for the zoea and megalopa stages, using an enriched diet such as artemia with HUFA (highly unsaturated fatty acids) to provide a high nutritional profile essential for growth and to establish a strong foundation for the immune system (Suprayudi *et al*. 2004).

This hybrid approach would be practical as it would leverages existing aquaculture practices, including quarantine and treatment, which are commonly practiced in broodstock management (Keenan & Blackshaw 2011). This method could reduce the duration required for broodstock conditioning compared to the time needed for breeding traditional SPF lines. This innovative strategy might offer significant cost-effectiveness and high scalability, potentially closing gaps in underdeveloped protocols, biosecurity challenges and economic barriers.

## CONCLUSION

Optimising broodstock management for mud crab is essential to improving the sustainability and productivity of tropical aquaculture, supporting coastal livelihoods and promoting environmental conservation through integration with mangrove ecosystem. However, challenges such as biosecurity risks and pathogen transmission have disrupted production consistency, particularly during the early stages from broodstock to larval rearing. Current practices, including broodstock selection based on ovarian maturation from wild-caught individuals, prophylactic formalin treatments and nutritional strategies, aim to mitigate these issues but also introduce risks, such as immune suppression and disease transmission from untreated feeds. The suggested hybrid strategy, which integrates the natural immune resilience of wild-caught broodstock with SPF-like condition, propose a practical solution for improving broodstock management. This approach involves implementing rigorous quarantine, disinfection, PCR screening and biosecure feeding strategies enriched with probiotics and nutritionally optimised diets. Together, these measures aim to mitigate pathogen risks while preserving innate immunity. This method not only targets existing biosecurity and economic challenges but also enhances reproductive performance and larval quality, laying the foundation for a more resilient and scalable mud crab aquaculture industry. Future research should focus on optimising formalin exposure, developing sterile feeds, and scaling SPF-like systems to ensure long-term sustainability and economic viability.

## Figures and Tables

**TABLE 1:** Common mud crab species used in hatcheries in different countries.

Country	Mud crab species used in hatcheries	Reference
China	*Scylla paramamosain*	(Han *et al*. 2024)
Vietnam	*S. paramamosain*	(Ngoc 2002)
Thailand	*S. paramamosain; S. olivacea*,	(Overton & Macintosh 2002; Oniam *et al*. 2025)
Malaysia	*S. paramamosain, S. olivacea, S. tranquebarica*	(Islam 2015; Asmat-Ullah *et al*. 2020; Shaiful Kahar *et al*. 2022)
Philippines	*S. serrata, S. tranquebarica and S. olivacea*	(Quinitio 2015)
Bangladesh	*S. serrata, S. olivacea*	(Hoq *et al*. 2014; Rouf *et al*. 2016)
